# Synergistic effects of psyllium husk powder and different levels of methylcellulose on the storage stability of sodium caseinate emulsion

**DOI:** 10.3389/fnut.2023.1125312

**Published:** 2023-02-09

**Authors:** Qing-quan Fu, Lei Zhou, Hai-bo Shi, Rong-rong Wang, Lin-wei Yang

**Affiliations:** ^1^School of Food Science, Nanjing Xiaozhuang University, Nanjing, Jiangsu, China; ^2^Key Laboratory of Meat Processing and Quality Control, Key Laboratory of Meat Processing, Jiangsu Synergetic Innovation Center of Meat Processing and Quality Control, Nanjing Agricultural University, Nanjing, China; ^3^School of Food Science and Engineering, South China University of Technology, Guangzhou, China; ^4^Nanjing Yurun Food Co., Ltd., Nanjing, China

**Keywords:** emulsion, carbohydrate, storage stability, rheology, microstructure

## Abstract

The study investigated the effects of compound fibers composed of psyllium husk powder (PHP, 0.3%) and methylcellulose (MC, 0, 0.3, 0.6, 0.9, and 1.2%) on the storage stability, rheology, and microstructure of sodium caseinate emulsions. Results showed that the emulsion stability was enhanced with the increased concentrations of MC, especially at the concentration of 1.2%. The oil droplet size in the emulsions was decreased as the concentrations of compound fibers increased, which was further confirmed by the optical microscope analysis. The rheological measurements and cryo-scanning electron microscopy results indicated that compound fibers improved the viscosity of the emulsions, and formed a strong three-dimensional network structure. The results of confocal laser scanning microscope and surface protein concentration measurements showed that compound fibers were evenly distributed into the oil droplet surface. The above results demonstrate that compound fibers are an effective thickener and emulsifier in enhancing the stability properties of oil-in-water (O/W) emulsions stabilized by sodium caseinate.

## 1. Introduction

Oil-in-water (O/W) emulsions are a mixed system where small spherical oil droplets (dispersed phase) are evenly distributed in water (continuous phase) through surfactant or surface-active polymers (1). O/W emulsions are widely applied in food, pharmaceutical, and cosmetic industries due to their excellent performance in delivering nutrients, and the crucial role as fat substitutes. However, O/W emulsions are unstable systems because of the occurrence of the separation of two phases induced by creaming, coalescence, flocculation, sedimentation as well as Ostwald ripening over a long period of time (2, 3). Hence, the addition of stabilizers is considered to be a valid method to enhance the storage stability of the O/W emulsions. The synthetic and natural stabilizers are commonly applied in food emulsions (4). With the development of human awareness for embracing health life, the natural stabilizers become more and more popular in the food industry (5). Protein and polysaccharides are the most commonly used natural stabilizer in food areas. Compared with protein stabilizers, polysaccharides are more prevalent for stabling food emulsion because they are more stable in different conditions such as pH, temperature, and ionic strength (6).

Psyllium husk powder (PHP) is abundant in complex polysaccharides, which is mainly composed of highly branched polysaccharides. Among these branched polysaccharides, the main chains and side chains are made up of xylopyranose residue, xylopyranose, and arabinofuranose residues, respectively (7). The polysaccharide PHP displays the increased viscosity and strong network structure against creaming, coalescence, flocculation, and thus enhances the storage stability of the emulsions (8). Therefore, PHP can be used as a functional ingredient in the preparation of various food products including bread, beverage, jams, health food, and meat products. Cellulose has been extensively used as food additives in recent decades since it provides health benefits for human. Methylcellulose (MC) is a water-soluble cellulose derivative, and it has been allowed to be extensively applied in food industry as an emulsifier, thickener and foam stabilizer (9, 10). MC can form highly viscous solution in the emulsions to retard the creaming or coalescence of the oil droplets, and finally improve the stability of the emulsions (11). Furthermore, MC contains much highly substituted regions by methyl groups (12), which endow MC to exhibit excellent surfactant properties and unique thermally reversible gelation during heating.

Sodium caseinate (NaCas) is a commercial product obtained by precipitating casein from milk. It is widely used to stabilize the emulsions as a thickener and emulsifier in the food areas (13). Generally, NaCas-stabilized emulsions are not stable at the unsuitable environmental conditions, such as freeze- thaw cycles, high ionic strength, and pH value close to the isoelectric point of caseinate (14, 15). Improved emulsion stability can be realized by the combination with compound fibers, which can surround the surface of oil droplets and enhance the viscosity of the emulsions. Previous reports have demonstrated that protein-polysaccharide complexes are beneficial to enhance the storage stability of O/W emulsions (16, 17). Based on the previous research, the appropriate proportion of compound fibers might help to improve the stability of protein stabilized emulsion. However, the experimental studies regarding to the storage stability of O/W emulsions prepared by compound fibers are still few. Therefore, the aim of the present research focused on investigating the influences of different levels of compound fibers on the stability, rheology, and microstructure of the emulsions.

## 2. Materials and methods

### 2.1. Materials and chemical reagents

Psyllium husk powder (PHP, protein content: 0.92%, fat content: 0.40%, total dietary fiber content: 87.30%, moisture content: 2.05%, ash content: 1.80%, calcium content: 0.11%, particle size: 100 mesh, purity: 99.88%) was purchased from Shanghai We Cheer Biotechnology Co., Ltd. (Shanghai, China). Methylcellulose (MC, A4M type, 4,000 cps of viscosity, 40 kD of molecular weight, degree of substitution: 1.6–2.0, moisture content: 1.58%, ash content: 1.26%) was supplied by Ashland Global Holdings, Inc. Food grade sodium caseinate was obtained from Jiangsu Yukun Biotechnology Co., Ltd. (Xuzhou, Jiangsu, China). Soybean oil was purchased from a local Haodi supermarket (Nanjing, China). Both Nile red and Nile blue were bought from Sigma-Aldrich Inc. (Shanghai, China). Other reagents used in current study were purchased from Shanghai Chemical Reagent Co., Ltd. (Shanghai, China).

### 2.2. O/W emulsion preparation

Soy oil (20%) and sodium caseinate (2%) were well blended using distilled water, and then the mixture was homogenized with an Ultra Turrax (IKA T25, Staufen, Germany) at 10, 000 r/min for 90 s to form a pre-emulsified solution. Next, PHP (0.3%) and MC (0, 0.3, 0.6, 0.9, and 1.2%) were added into the above homogenate solution at the same time, and then homogenized again with the same speed and time. At the same time, the pre-emulsified solution without PHP and MC was regarded as the control sample. Therefore, the total concentration of the compound fibers made of PHP and MC was 0, 0.3, 0.6, 0.9, 1.2, and 1.5%, respectively. The emulsions were emulsified by a homogenizer twice, and then used to measure different indicators.

### 2.3. Observation of visual appearance

The visual appearances of the emulsions were observed following the method described by Fu et al. (18). The prepared emulsions were promptly divided equally into the glass tubes equipped with screw caps, and then stored at 4°C for 2 h and 7 d, respectively. The creaming phenomenon of emulsions was observed and photographed using a phone camera (iPhone 12, Apple, USA).

### 2.4. Turbiscan stability index (TSI)

The TSI values of the emulsions (20 mL) were measured using a Turbiscan Lab Expert analyzer (Formulaction, Toulouse, France) according to the method of Wang et al. (19). The emulsion was transferred to a cylindrical glass cell up to the height of holder, and scanned by a pulsed near infrared (wavelength 880 nm) light source. The total time of the scanning and one scan time of the analyzer at room temperature were set as 4 h and 60 s, respectively. The images of the TSI were acquired by the Turbiscan Easysoft Lab 2.0 software.

### 2.5. Emulsion activity index and emulsion stability index

The emulsion performance index of the emulsions was determined according to Zhou et al. (20) with minor modifications. The samples (50 μL) were added with 5 mL of 0.1% sodium dodecyl sulfate solution, and the absorbance at 0 and 10 min was measured at 500 nm after being well mixed. The emulsion activity index (EAI) and emulsion stability index (ESI) values were calculated by the following equations, respectively.


$$E\,A\,I\,\left(m^{2}/g\right)=2\times 2.303\times \frac{{\text{A}}_{0}\times \text{DF}}{C\times \left(1-\varphi \right)\times 10^{4}}$$

$$ESI\left(\%\right)=\frac{A_{10}}{A_{0}}\times 100$$

Where A_0_ and A_10_ represent the absorbance measured at 0 min and 10 min, respectively; C represents the concentration (g/mL) of sodium caseinate; φ is the oil volume fraction (v/v) of emulsions, and D represents the dilution factor.

### 2.6. Droplet size and zeta potential (ζ-potential)

The size of oil droplets were determined with a 3,000 Mastersizer instrument (Malvern Instruments Inc., UK) according to Zhang et al. (21) with a slight modification. Before measurement, the Mastersizer instrument was equilibrated at 25°C for 10 min, and the refractive index and absorption coefficient were set as 1.52 and 0.001, respectively. The prepared emulsions were diluted to 1 mg/mL with distilled water, and the ζ-potential were measured with a Nano-ZS90 Zetasizer (Malvern Instruments Ltd., Nottinghamshire, UK).

### 2.7. Rheology measurement

The storage modulus (G′) and loss modulus (G″) values of the emulsions were measured according to the method of Hu et al. (6). A MCR301 rheometer (Anton Paar, Austria, with 0.5 mm gap and P50 parallel plate) was used to evaluate the rheology, and the measurements were conducted within 0.1–10 rad/s. For apparent viscosity scanning with the shear rate ranged from 0.1 to 100 s^–1^.

### 2.8. Optical microscopy

The microstructure of oil droplets in the emulsions were determined by an optical microscope (BH2-UMA, Olympus Corporation, Tokyo, Japan) with 50-fold eyepiece, and the images were taken at room temperature.

### 2.9. Observation of confocal laser scanning microscope (CLSM)

The distribution and the size of the emulsion oil droplets and proteins were observed with a FV300 high-resolution CLSM (Olympus, Tokyo, Japan). The prepared emulsions (1 mL) were dyed for 20 min using the mixture of Nile red (20 μL, 0.1%, w/v) and Nile blue (20 μL, 0.1%, w/v) under dark conditions (22). The excitation wavelength of fluorescent dyes in the emulsions were 488 nm (Nile red) and 633 nm (Nile blue), respectively.

### 2.10. Cryo-scanning electron microscopy (Cryo-SEM)

The surface distribution and structure of compound fibers in the emulsions were observed at 2,000 × using a SU8010 Cryo-SEM (Hitachi, Tokyo, Japan). The fresh emulsions were diluted 10 times with distilled water, and 2 mL of diluted samples was frozen in liquid nitrogen for 15 min. The picture was immediately captured at an acceleration voltage of 5.0 kV.

### 2.11. Surface protein content

Surface protein contents of the emulsions were determined according to the method of Zhao et al. (17). An aliquot of the emulsions was centrifuged at 10,000 × *g* for 60 min at 20°C, and each supernatant was then taken out and filtered with a 40 μm filler after the centrifugation. Finally, the protein content of the emulsions was determined by a K9860 Kjeldhal apparatus (Haineng, Shandong, China), and the surface protein content was calculated using the following equation.


$$\mathrm{Surface}\,\mathrm{protein}\,\mathrm{content}\,\left(\mathrm{mg}/m^{2}\right)=$$

$$\frac{\mathrm{Total}\,\mathrm{protein}\,\mathrm{content}-\mathrm{subnatant}\,\mathrm{protien}\,\mathrm{contentpyrene}}{\mathrm{specific}\,\mathrm{surface}\,\mathrm{area}}\times 1000$$

Where total protein content is the protein content used to fabricate emulsions and specific surface area is acquired by Mastersizer 3,000.

### 2.12. Statistical analysis

All the measurements were performed three times, and the results were expressed as the mean values ± standard deviations (SD). A Statistical Analysis System (SAS) version 8.0 (Institute Inc., USA) was used to analyze the difference (one-way-ANOVA) of the related data. Differences between means were detected by Duncan’s multiple range test, and considered at a significance of *P* < 0.05.

## 3. Results and discussion

### 3.1. The creaming stability, TSI values and emulsion performance indexes of the O/W emulsions

**FIGURE 1:**
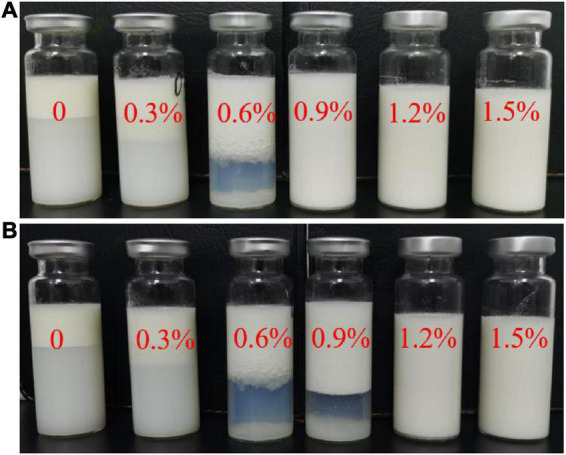
The visual observations of emulsions with different concentrations of compound fibers. **(A,B)** Represent the emulsions after storage at 4°C for 2 h and 7 d, respectively.

Creaming stability is an extremely important indicator which reflects the coalescence and separation state of emulsions. Effects of different levels of compound fibers on visual appearance in the emulsions during a short-term storage (up to 7 days) are shown in Figure 1. The emulsions prepared without compound fibers (control) showed an obvious creaming phenomenon within 2 h (Figure 1A), and the creaming slowly reduced with the incremental concentration of compound fibers, but no obvious water layer was found as the concentration of the compound fibers reached 0.9%. After storage for 7 days (Figure 1B), four levels of the emulsions (0, 0.3, 0.6, and 0.9%), expect for 1.2 and 1.5% groups, exhibited creaming phenomena. These changes indicated that the emulsion storage stability was significantly improved with the incremental concentration of compound fibers. Furthermore, owing to the hydrophilic groups of dietary fiber (PHP and MC), the bonding of water molecules in the capillaries of protein matrix was enhanced with PHP and MC addition, consequently improving the volume of creaming phase in the emulsion system (23). As exhibited in Figure 1, the emulsions with lower incorporation of 0 and 0.3% compound fibers showed a turbid serum phase, which might be attributed to the addition of fibers could enhance the surface protein content in the creaming phase (24). Proper level of compound fibers was able to stabilize the NaCas-stabilized emulsions, and to prevent the creaming due to the thickening properties and the formation of gel-like network structure. The study of Jia et al. (25) also demonstrated that amorphous cellulose could effectively stabilize the emulsions by forming the network structure.

**FIGURE 2:**
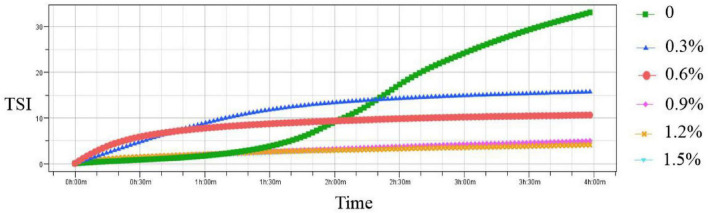
The TSI values of the fresh emulsions with different concentrations of compound fibers.

To further investigate the physical stability of emulsions, TSI values of the emulsions were measured in the current study. The TSI value is used to characterize the migration and interaction of particles using multiple light scattering (26). Figure 2 shows the addition of different levels of compound fibers on the stability of the emulsions. Compared with the control group, the TSI values in the emulsions with compound fibers were slowly decreased with the prolongation of the test time indicating that the emulsion stability was getting better. Moreover, the emulsion stability could be improved by increasing the concentrations of the compound fibers. The above results might be due to the interaction between MC and PHP leading to the formation of the gel-like network structure which enhanced the viscosity of the emulsions (27). In addition, proper addition of PHP (0.3%) in current study was beneficial to further enhance the NaCas-based emulsions by impeding the movement of oil droplets (28). The result was similar with the tendency of Xu et al. (29) who found that TSI values in the cyclodextrin-based emulsions decreased with the incremental concentrations of MC.

**FIGURE 3:**
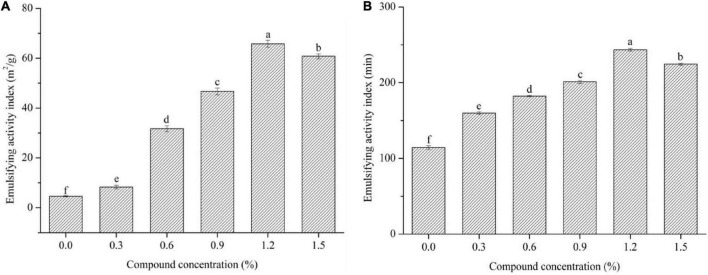
The EAI **(A)** and ESI **(B)** values of the emulsions with different levels of compound fibers. The data are expressed as the mean ± standard error. Values with different lowercase letters are significantly different (*P* < 0.05).

Emulsion activity index and ESI values are usually to characterize the ability of surface protein contents absorbed at the oil-water interface in the emulsions to prevent creaming, flocculation, and coalescence, and to keep the stability of the emulsions during storage, respectively (12). As displayed in Figure 3, the increased concentration of the compound fibers significantly increased the EAI and ESI values of the emulsions compared to the control group (*P* < 0.05). However, the EAI and ESI values reached the maximum with the incorporation of 1.2% compound fibers, and then decreased when the compound concentration further increased (1.2% MC + 0.3% PHP). Similar result was reported by Li et al. (30), where the structure of MC in compound matrix became less ordered as concentration was higher than 1.0%, and thus slightly decreased the emulsion stability. The above results indicated that the addition of different levels of compound fibers could effectively boost the storage stability of the emulsions, which may be due to the uniform distribution between the proteins and oil droplets caused by the viscosity of compound fibers.

### 3.2. Droplet size distribution and ζ-potential of emulsions

**FIGURE 4:**
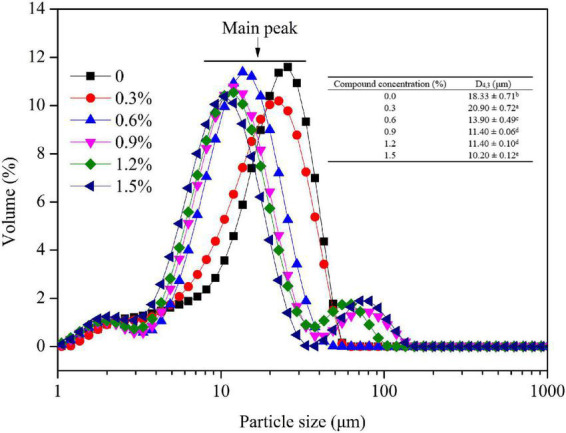
Droplet size of the emulsions with different concentrations of compound fibers. The data are expressed as the mean ± standard error. Values with different lowercase letters are significantly different (*P* < 0.05).

The droplet size is a key factor influencing the emulsion stability by functioning on the creaming rate (31). As shown in Figure 4, the main peak of other groups was transferred to the left compared to control group, suggesting that the introduction of compound fibers promoted the formation of small oil droplets. The small size of emulsions significantly increases Brownian motion thereby overcoming gravitational forces and reducing sedimentation and creaming during storage (32). Meanwhile, the mean droplet size of emulsions was decreased from 18.33 μm to 10.20 μm, manifesting better dispersion and stability of oil droplet. MC, as an emulsifier, can also create an interfacial coating/layer on the droplets and produce steric or electrostatic repulsive forces that prevent the aggregation of emulsified droplets. These results might be attributed to the interaction between MC and proteins, and the increased viscosity with the addition of PHP (33). The result was consistent with the microstructure of oil droplets observed by optical microscope, CLSM, and cryo-SEM. Gullapalli and Sheth (34) reported that the oil droplet size in the lipophilic emulsifier emulsions significantly decreased as MC concentration increased.

**FIGURE 5:**
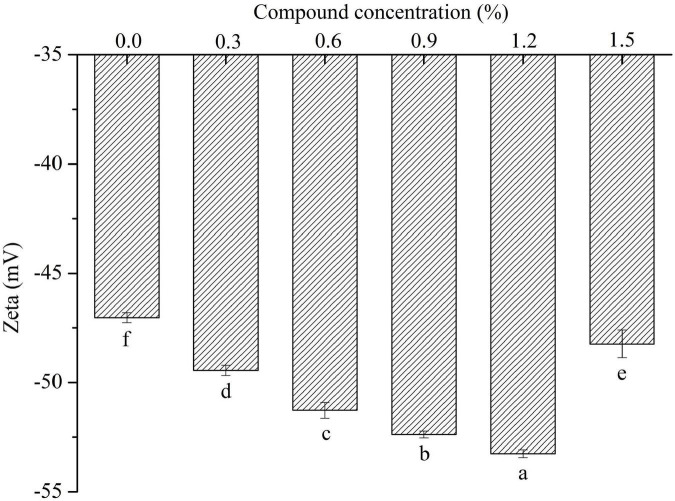
The zeta potential of the emulsions with different concentrations of compound fibers. The data are expressed as the mean ± standard error. Values with different lowercase letters are significantly different (*P* < 0.05).

The change of surface charge density is an evidence of electrostatic interaction between protein and polysaccharide molecules in the emulsion system (35). The ζ-potential results (Figure 5) showed that all the experiments were negative at ζ-potential value, and demonstrated that the emulsion stabilized by sodium caseinate had high electrostatic repulsion. It was observed that the ζ-potential significantly decreased with the increased concentration of compound fibers, indicating that the emulsions more negative charges were determined, which might be generated by the addition of the compound fibers. The compound fibers with negative charges were homogeneously distributed at the oil-water interface, and prevented the creaming and coalescence of the oil droplets as reported by Zhao et al. (36).

### 3.3. Rheological properties

**FIGURE 6:**
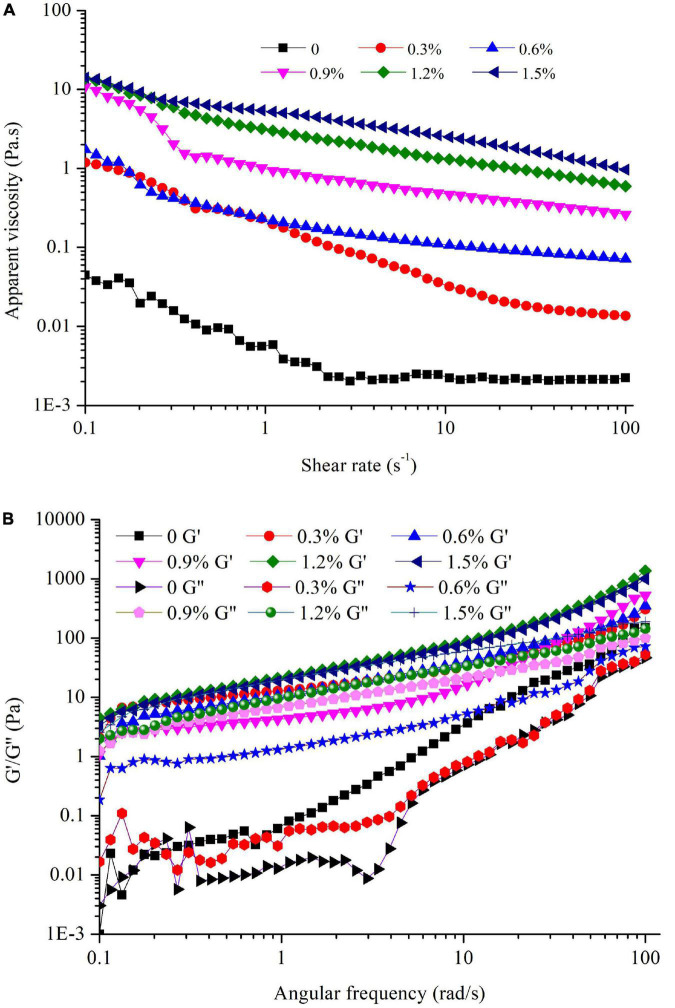
The apparent viscosity **(A)**, G′ and G″ values **(B)** of the emulsions with different concentrations of compound fibers.

The apparent viscosity of the emulsions containing different concentrations of compound fibers is shown in Figure 6A. Regardless of the compound fibers concentration, the viscosity of the emulsions was significantly decreased with increased shear rate, manifesting the shear thinning behavior and non-Newtonian properties of the samples. The shear thinning property is typically characterized by the reduction of the flocculated oil droplets as the shear rate increased (37). Moreover, we could observe that the viscosity of the emulsions was increased as the concentration of compound fibers increased. The result demonstrated that compound fibers increased the viscosity of the emulsions by acting as a thickening agent, and limited the creaming or coalescence of the oil droplets. On the one hand, the occurrence of PHP could improve the shear thinning characteristics of the continuous phase, making the oil droplets easier to disperse (28). On the other hand, MC acted to thicken or form a gel-like droplet network, thereby delaying the movement of the droplets to prevent aggregation. The compound fibers with high hydrophilicity and high viscosity thus changed the rheological behavior of the emulsions (38, 39). These findings are also in good agreement with the results of visual appearance, TSI and ESI values. The increased viscosity can effectively inhibit the movement of oil droplets and further reduce the creaming and coalescence of the emulsions. Our results are consistent with the results of Hu et al. (6) and Jia et al. (25) who reported that regenerated cellulose increased the viscosity of emulsions and improved the stability of emulsions.

The viscoelastic properties of the emulsions with different levels of compound fibers were presented in Figure 6B. The G′ and G″ values of the emulsions were increased as angular frequency increased regardless of the compound fibers concentration. Furthermore, G′ and G″ values increased as the concentration of compound fibers was augmented, indicating that the addition of compound fibers had an important and positive impact on the structure of the emulsions. MC is a type of cellulose-based derivatives, in which methyl groups are covalently linked to the cellulose main chains, conferring MC with remarkable viscosity properties (30). From Figure 6B, it could be seen that G′ values was higher than G″ values, and no crossover occurred with the increment of the angular frequency, demonstrating the elastic gel-like properties (40). These results agreed with the report of previous studies, and they demonstrated that the G′ was higher than G″ value in the emulsions prepared with myofibrillar proteins and lard oil or regenerated cellulose (17, 41).

### 3.4. Microstructure analysis

**FIGURE 7:**
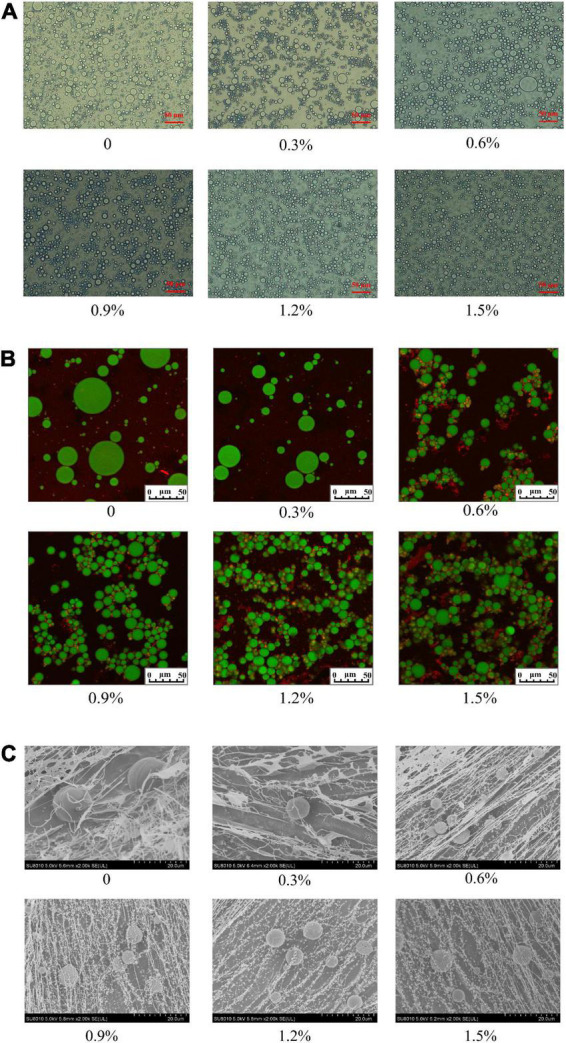
The microstructure of the emulsions with different concentrations of compound fibers. Optical **(A)**, CLSM **(B)** and cryo-SEM **(C)**. The scale bar represents 50 μm.

The results of the optical observations are presented in Figure 7A. The oil droplets of the emulsions became smaller and uniform with the incremental concentrations of compound fibers, suggesting that that the emulsions with compound fibers were relatively stable at higher concentration. The mechanism of the stabilization of the emulsions might be ascribed that the increased viscosity properties mediated by adding MC which delayed or retarded the mobility of oil droplets. The result agreed with the findings of the oil droplets observed by using CLSM and cryo-SEM. Our results showed similar tendency to the result reported by Xu et al. (29), who demonstrated that the oil droplets of the cyclodextrin-based emulsions decreased as the concentration of MC augmented.

The CLSM is an effective method to intuitively illustrate the emulsion profile of the emulsions. It can be found that the oil droplet sizes were decreased with the increment of the concentration of compound fibers (Figure 7B). The size of oil droplets of the emulsion prepared with 1.2% of compound fibers was smallest, and evenly more uniform compared to the control and other groups. In addition, we also observed that the proteins partly wrapped the oil droplets after the addition of higher concentration of compound fibers (0.9–1.5%). This might be due to the fact that the addition of compound fibers promoted the interactions between proteins and oil droplets. Our results are in good agreement with Xu et al. (29), who proved that MC surrounded the oil droplets of the emulsions by forming a strong network structure against the creaming and coalescence. Meanwhile, the unabsorbed MC increased the viscosity of emulsions and limited the coalescence or flocculation of oil droplets.

To further investigate the interactions between the compound fibers and the oil droplets in the emulsions, we used cryo-SEM to visualize the oil droplets and compound fibers in the emulsions. It can be clearly observed in Figure 7C, a network structure was arranged in order, and the structure became denser with the increased compound fibers concentration. Remarkably, the emulsions with 1.2 and 1.5% compound fibers were highly dense, and exhibited no creaming phenomena during the storage. The cryo-SEM micrographs also showed that the size of oil droplets was decreased with the increased of the compound fibers concentration. The result showed similar trends with the findings of particle size, optical micrographs, and CLSM. In addition, from the cryo-SEM micrographs it could be seen that the compound fibers were absorbed on the surface of oil droplets. Therefore, we speculated that compound fibers had amphiphilic properties, and formed the network structure to stabilize the O/W emulsions.

### 3.5. The surface protein content of the emulsions

**FIGURE 8:**
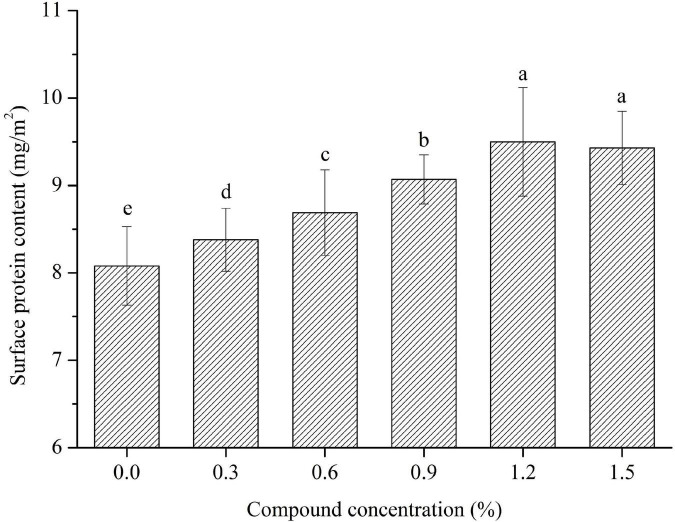
The surface protein content of the emulsions with different concentrations of compound fibers.

PHP also contain amounts of proteins (about 0.92% of total PHP) besides the sodium caseinate. The surface protein content of the emulsions is shown in Figure 8, and it is significantly affected by the concentration of compound fibers. The absorbed protein content obviously increased with the increase of the concentration of compound fibers, whereas the number of interfacial proteins were improved from 8.08 mg/cm^2^ to 9.50 mg/cm^2^ with the increased addition of compound fibers concentration from 0 to 1.2%. The possible reason for this result is that the addition of compound fibers increased the viscosity properties, and formed the gel network structure around the oil droplets. However, the surface protein content of the emulsions prepared with 1.5% had no significant difference compared with sample containing 1.2% compound fibers. The above basic reason is that the amounts of absorbed proteins increased as the concentration of compound fibers were increased from 1.2 to 1.5%, while special surface area actually decreased. Our findings have similar trends with the results of Hu et al. (6) and Zhao et al. (17) who reported that the amount of surface proteins significantly increased as the concentration of regenerate cellulose in the emulsion increased. Overall, the compound fibers improved the protein absorption and therefore is beneficial to the stability of the emulsions.

## 4. Conclusion

Adding a composite fiber composed of PHP and MC are beneficial to the storage stability of emulsions due to their good thickening ability. The results of visual observation, TSI, EAI and ESI values demonstrated that the stability of the O/W emulsions was significantly influenced by the concentration of compound fibers. The prepared O/W emulsions displayed the synergistic effects on the physical stability, the size of oil droplets and rheological properties after the mixed compound fibers were incorporated. The concentration of the compound fibers 1.2% incorporated into the O/W emulsions enhanced the gel-forming network structure and thus inhibited the creaming and coalescence due to its good thickening and gelling properties. In addition, compound fibers could adsorb and wrap evenly the surface of the oil droplets, and correspondingly reduced the size of the oil droplets (from 18.33 μm to 10.20 μm) and prevented the flocculation of the oil droplets. At the same time, the numbers of interfacial proteins were improved from 8.08 mg/cm^2^ to 9.50 mg/cm^2^ as the concentration of the compound fibers incremented. The above results could be the possible mechanism that compound fibers stabilized the O/W emulsions formed by sodium caseinate. Further research should be done to investigate the application of the O/W emulsions stabilized by sodium caseinate as a fat substitute in the meat industry.

## Data availability statement

The raw data supporting the conclusions of this article will be made available by the authors, without undue reservation.

## Author contributions

Q-QF: conceptualization, data curation, writing—review and editing, and project administration. LZ and H-BS: methodology and writing—review and editing. R-RW and L-WY: formal analysis and writing—review and editing. All authors contributed to the article and approved the submitted version.
